# Supplementary material for: Deep Learning Approaches to Osteosarcoma Diagnosis and Classification: A Comparative Methodological Approach
Source: Cancers (Basel). 2023 Apr 13;15(8):2290. doi: 10.3390/cancers15082290 (PMC10136449; doi:10.3390/cancers15082290)
Supplement: Supplementary file 1 [file cancers-15-02290-s001.zip › Figure S1 .pdf]

Article

Deep Learning Approaches to Osteosarcoma Diagnosis and Classification: A Comparative Methodological Approach

Ioannis A. Vezakis [1], George I. Lambrou [1,2,3] and George K. Matsopoulos [1,*]

[1] Biomedical Engineering Laboratory, School of Electrical Engineering, National Technical University of Athens, Athens, Greece
[2] Choremeio Research Laboratory, First Department of Pediatrics, National and Kapodistrian University of Athens, Thivon & Levadeias 8, 11527 Athens, Greece
[3] University Research Institute of Maternal and Child Health & Precision Medicine, National and Kapodistrian University of Athens, Thivon & Levadeias 8, 11527 Athens, Greece
* Correspondence: G.K.M. gmatsopoulos@biomed.ntua.gr

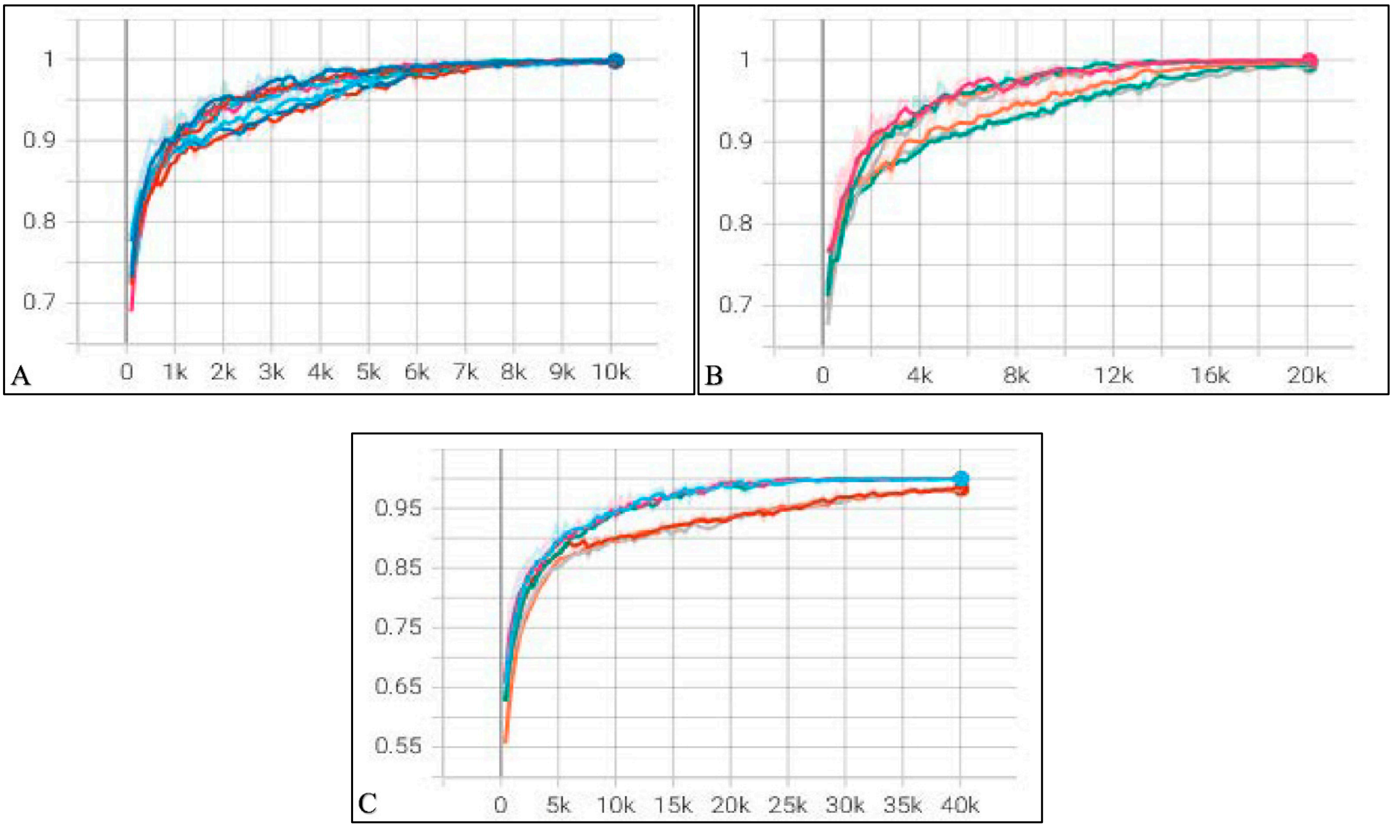

Figure S1. The overall training accuracies, plotted over 100 epochs, superimposed for all networks with input image size: **A**) 256x256; **B**) 512x512; **C**) 1024x1024. In all cases, the training accuracy tends to approach a perfect score. VGG networks have been excluded as they failed to learn in our experiments.
