# Supplementary material for: Deep Learning Approaches to Osteosarcoma Diagnosis and Classification: A Comparative Methodological Approach
Source: Cancers (Basel). 2023 Apr 13;15(8):2290. doi: 10.3390/cancers15082290 (PMC10136449; doi:10.3390/cancers15082290)
Supplement: Supplementary file 1 [file cancers-15-02290-s001.zip › Table S1 .pdf]

Article

Deep Learning Approaches to Osteosarcoma Diagnosis and Classification: A Comparative Methodological Approach

Ioannis A. Vezakis [1], George I. Lambrou [1,2,3] and George K. Matsopoulos [1,*]

1 Biomedical Engineering Laboratory, School of Electrical Engineering, National Technical University of Athens, Athens, Greece
2 Choremeio Research Laboratory, First Department of Pediatrics, National and Kapodistrian University of Athens, Thivon & Levadeias 8, 11527 Athens, Greece
3 University Research Institute of Maternal and Child Health & Precision Medicine, National and Kapodistrian University of Athens, Thivon & Levadeias 8, 11527 Athens, Greece
* Correspondence: gmatsopoulos@biomed.ntua.gr

"Macro-averaging reduces the multiclass predictions down to multiple sets of binary predictions, calculates the corresponding metric for each of the binary cases, and then averages the results together" (https://cran.r-project.org/web/packages/yardstick/vignettes/multiclass.html, accessed March 8th, 2023).

As our investigation concerned a multiclass case (i.e. normal tissue, tumors (osteosarcoma) and necrotic tissue) we have selected *F*1 macro-averaging in order to reduce the problem to "multiple one-vs-all comparisons". Macro-averaging is performed using the following equation:

$$F1_{Macro} = \frac{F1_1 + F1_2 + \ldots + F1_k}{k} = F1_1\frac{1}{k} + F1_2\frac{1}{k} + \ldots + F1_3\frac{1}{k} \quad (1)$$

In macro averaging, all classes obtain an equal weight, when contributing their portion of the precision value to the total.

Supplementary Table S1. *F*1 scores for each network and image size.

Network 1	Image Size	F1 Score			
		Non-Tumor	Viable Tumor	Necrosis	Macro-Averaged *F*1 Score
MobileNetV2	256×256	0.94	0.89	0.85	0.893
EfficientNetB0	256×256	0.95	0.87	0.85	0.890
EfficientNetB3	1024×1024	0.94	0.89	0.84	0.890
ResNet34	512×512	0.93	0.92	0.82	0.890
EfficientNetB0	1024×1024	0.93	0.89	0.84	0.887
EfficientNetB7	512×512	0.94	0.88	0.84	0.887
ResNet34	256×256	0.92	0.92	0.82	0.887
EfficientNetB1	256×256	0.95	0.86	0.84	0.883
EfficientNetB7	256×256	0.95	0.87	0.83	0.883
ResNet50	256×256	0.94	0.89	0.82	0.883
EfficientNetB0	512×512	0.93	0.88	0.83	0.880
EfficientNetB1	512×512	0.94	0.88	0.82	0.880
VGG16	256×256	0.93	0.89	0.81	0.877
EfficientNetB5	896×896	0.92	0.89	0.81	0.873
EfficientNetB5	512×512	0.93	0.87	0.82	0.873
ResNet50	512×512	0.92	0.88	0.82	0.873
EfficientNetB3	256×256	0.93	0.87	0.81	0.870
ResNet18	256×256	0.92	0.88	0.81	0.870
EfficientNetB1	1024×1024	0.93	0.85	0.82	0.867
EfficientNetB3	512×512	0.93	0.86	0.81	0.867
EfficientNetB5	256×256	0.94	0.84	0.80	0.860
MobileNetV2	512×512	0.92	0.85	0.81	0.860
ResNet50	896×896	0.90	0.89	0.77	0.853

ResNet18	512×512	0.92	0.85	0.78	0.850
ViT-B/16	224×224	0.88	0.83	0.72	0.810
ResNet18	1024×1024	0.83	0.86	0.72	0.803
ResNet34	1024×1024	0.82	0.87	0.70	0.797
MobileNetV2	1024×1024	0.82	0.84	0.66	0.773
VGG16	1024×1024	0.63	-	-	0.630
VGG16	512×512	0.63	-	-	0.630
VGG19	896×896	0.63	-	-	0.630
VGG19	512×512	0.63	-	-	0.630
VGG19	256×256	0.63	-	-	0.630
